# Immune Microenvironment in Langerhans Cell Histiocytosis: Potential Prognostic Indicators

**DOI:** 10.3389/fonc.2021.631682

**Published:** 2021-05-07

**Authors:** Chuchu Feng, Yang Li, Huang Ke, Xiaomin Peng, Haixia Guo, Liping Zhan, Xilin Xiong, Wenjun Weng, Jiaqiang Li, Jianpei Fang

**Affiliations:** ^1^ Department of Pediatric Hematology/Oncology, Sun Yat-Sen Memorial Hospital, Sun Yat-Sen University, Guangzhou, China; ^2^ Department of Pediatrics, Nanfang Hospital, Southern Medical University, Guangzhou, China

**Keywords:** Langerhans cell histiocytosis, tumor immunology, children, cytokines, inflammation

## Abstract

In this study, the immune microenvironment in Langerhans cell histiocytosis (LCH) was characterized to determine if immune indices are predictive of severity. Serum samples from 54 treatment-naïve patients were analyzed quantitatively for inflammatory cytokines and immunoglobulins before and after the induction of chemotherapy. The initial serum sIL-2R, TNF-α, and IL-10 of untreated LCH patients with risk organ involvement (RO+) were significantly higher than those with single-system (SS) involvement. LCH patients with hematologic involvement exhibited a significantly higher sIL-2R, TNF-α, IL-10, and IL-1β expression, as compared to the group without involvement. sIL-2R, TNF-α, and IL-10 were increased in patients with liver or spleen involvement. Th cells have decreased in the liver+ and spleen+ group, and Ts cells were significantly decreased in non-response group after induction chemotherapy. The serum level of immune indices represents, to some extent, the severity of the disease. Pertinent laboratory inspections can be used to improve risk stratification and guide immunotherapy.

## Introduction

Langerhans cell histiocytosis (LCH) is a histiocytic disorder arising from the mononuclear phagocyte system, which results in the abnormal accumulation and proliferation of LCH cells. LCH is more common in children than in adults, with the clinical manifestations varying from isolated osseous, mucocutaneous, and pulmonary involvement to multi-system (MS) involvement, such as lymph node, bone marrow, liver, spleen, gastrointestinal tract, thymus, endocrine gland, and central nervous system involvement, causing hyperplasia, fibrosis, necrosis and other pathological changes, eventually leading to organ dysfunction (1). In 2010, Badalian-very found 57% of BRAF V600E gene positive-mutation in 61 LCH patients (2). The disease is an inflammatory myeloid neoplasia with the characteristics of both an abnormal reactive process and a neoplastic process. Subsequent studies showed that the mutation rate of BRAF gene in LCH cases was 45% to 65%, suggesting that the BRAF V600E gene was closely related to LCH incidence (3–11). De Graaf et al. found a variety of cytokines expressed in LCH lesions, such as IL-1, TGF-α, TGF-β, TNF-α, and TFN-γ (11). Kannourakis et al. extracted and cultured monocytes from eosinophilic granulomatous tissues in patients with LCH, and found that such monocytes could highly express IL-1, TNF-α, GM-CSF, IL-8, and LIF-21 (12). In addition to the “cytokine storm” of local lesions, the apparent level of some inflammatory cytokines in the serum of LCH patients increased, suggesting that cytokines may be associated with the pathogenesis of LCH. At present, the etiology and pathogenesis of LCH remains speculative, bringing about uneven curative effect and lacking effective prognosis indicator. In the present study, the immune function of LCH children admitted to our hospital in the past 7 years was reviewed before and after induction treatment. Clarifying the immune status of LCH children will help provide insights into LCH prognosis and, ultimately, optimize and personalize therapy.

## Methods

### Patients

This study was performed at the Sun Yat-sen Memorial Hospital (Guangzhou, China) between March 2013 and September 2020. A total of 54 children (37 males and 17 females; median age, 3.6 years; age range, 2.0 months to 12.0 years) were enrolled in this study. All patients fulfilled accepted diagnostic criteria established by the Histiocyte Society in 2009. Evaluation after the induction chemotherapy of JLSG-96/02 or Chinese Children’s Histiocytic Group (CCHG)-LCH-2019 regimens was performed based on the following criteria (13): i) non active disease (NAD), no evidence of disease, Resolution of all signs or symptoms; ii) active disease-better (AD-B), regression of signs or symptoms, no new lesions; iii) active disease-mixed (AD-I), new lesions in one site, regression in another site; iv) active disease-stable (AD-S), persistence of signs or symptoms, no new lesions; vi) worse, progression of signs or symptoms and/or appearance of new lesions. In isolated bone disease progression is defined as appearance of new bone lesions or lesions in other organs. Risk organs include the hematologic system, the spleen and the liver. All methods were carried out in accordance with relevant guidelines and regulations. All peripheral blood samples were obtained with written informed consent from the legal guardian of patients. The study was approved by the ethics committee at Sun Yat-sen Memorial Hospital.

### Cytokine, Immunoglobulin, and Lymphocyte Subset Measurement

Prior to treatment, and 2 weeks after the initial induction of chemotherapy, the serum levels of the cytokines IL-8, IL-6, IL-10, IL-1β, and sIL-2R were measured using the IMMULITE-1000 Immunoassay System (Siemens Healthineers, Erlangen, Germany) while the immunoglobulins IgA, IgG, IgM and IgE were detected by the BN II system (Siemens Healthineers). Lymphocyte subsets in the peripheral blood, including T, B, natural killer, cytokine-induced killer, T helper (Th), suppressor T (Ts), Th/Ts, and regulatory T cells (Treg) were tested using a FACSCanto™ II flow cytometer (Beckman Coulter, Inc., Brea, CA, USA), using a BD Multitest™ 6‐color TBNK kit and DIVA software.

### Statistical Analysis

Data were statistically analyzed using SPSS software (version 21.0). The continuous variables were expressed as the mean ± standard deviation and the count variables were expressed in frequency/rate. Original data of inflammatory factors IL-10 and IL-1β were expressed as <5 pg/ml, which were transformed into qualitative variables in the correlation analysis. The non-paired t and Kruskal-Wallis H tests were used to assess the statistical significance between the groups. To compare qualitative data we used the Fisher’s exact test. P<0.05 was considered to indicate a statistically significant difference.

## Results

*Patient Characteristics* (**Table 1**).

**Table 1:** Clinical characteristics of 54 children with LCH.

	No. of patients
Gender	Gender
Male	37
Female	17
Age at diagnosis (yrs)	
Median	3.6
Disease classification	
Multifocal single system (SS)	17
Multiple system without risk organ involvement (MS-RO-)	15
Multiple system with risk organ involvement (MS-RO+)	22
Risk organ involvement	
Liver	18
Spleen	6
Hematologic	3
Response at week 6	
NAD	9
AD-B	33
AD-I	1
AD-S	8
Worse	3

### Analysis of Immune Indices and Clinical Types

**Figure 1:**
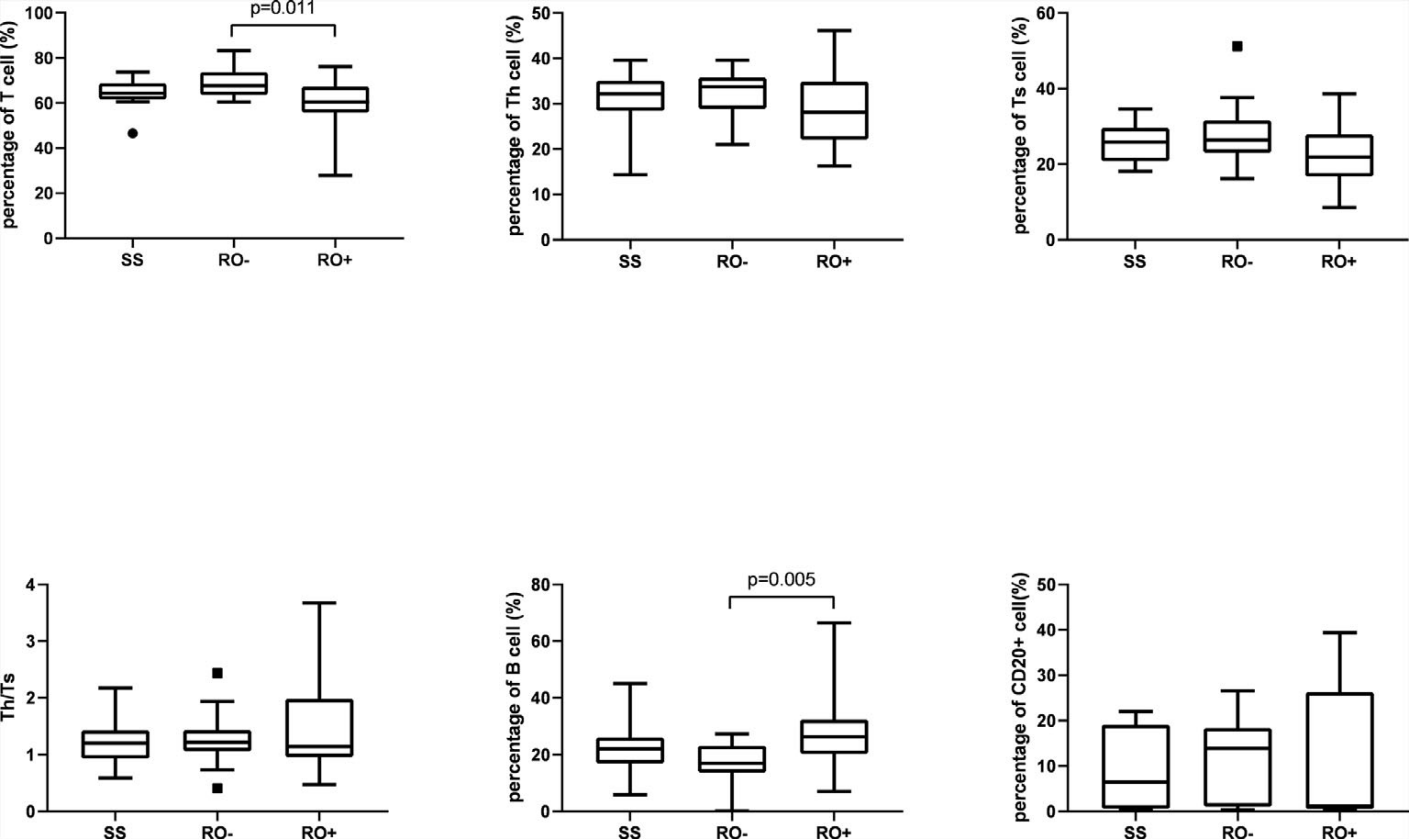
The serum level of inflammatory cytokines in disease classification.

**Figure 2:**
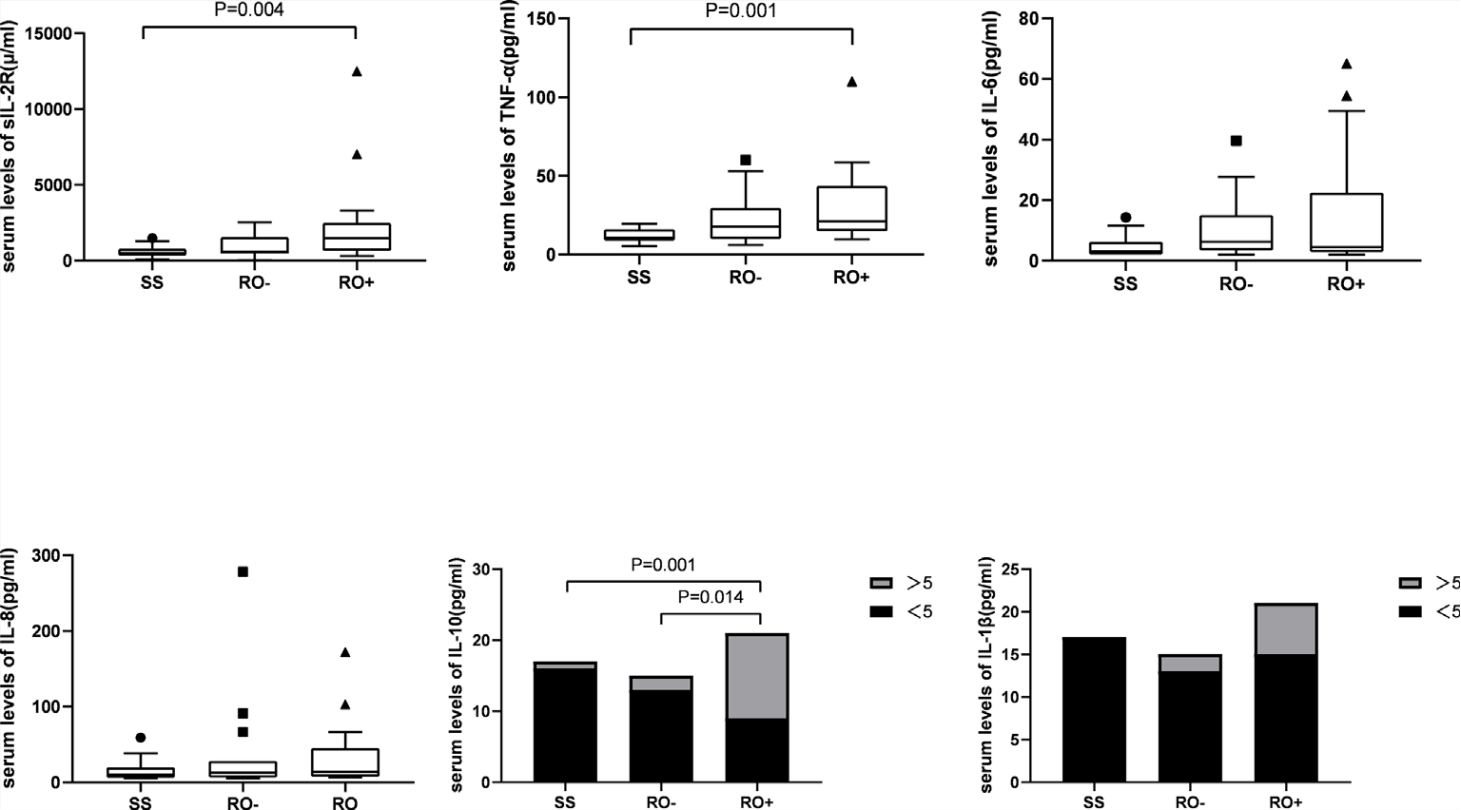
The lymphocyte subset in disease classification.

A total of 17 patients had multifocal single system disease (SS), 15 patients had multiple system disease without risk organ involvement (RO-), and the remaining 22 patients had risk organ involvement (RO+). Among the inflammatory cytokines, as shown in **Figure 1**, significantly higher serum levels of sIL-2R (2244.2 ± 2790.9 *vs* 595.4 ± 366.7 μ/ml), TNF-α (31.0 ± 24.0 *vs* 11.8 ± 4.6 pg/ml), and IL-10 were observed in the RO+ group, as compared to the SS group. The percentages of T cells in peripheral blood were obviously lower in the RO+ group than RO-, while B cells obviously increased (**Figure 2**). No statistical significance was identified in immunoglobulin (**Table 2**).

**Table 2:** Immunoglobulin in different disease classification.

Immune indices	Disease classification			
	SS	MS-RO-	MS-RO+	P-value
IgA(g/l)	2.0 ± 1.0	1.2 ± 0.7	1.4 ± 1.0	0.052
IgG(g/l)	12.5 ± 2.9	10.3 ± 3.6	10.6 ± 3.5	0.178
IgM(g/l)	1.5 ± 0.5	1.3 ± 0.5	1.4 ± 0.7	0.729
IgE(IU/ml)	543.4 ± 1275.1	166.3 ± 284.0	152.6 ± 226.8	0.466

**Figure 3:**
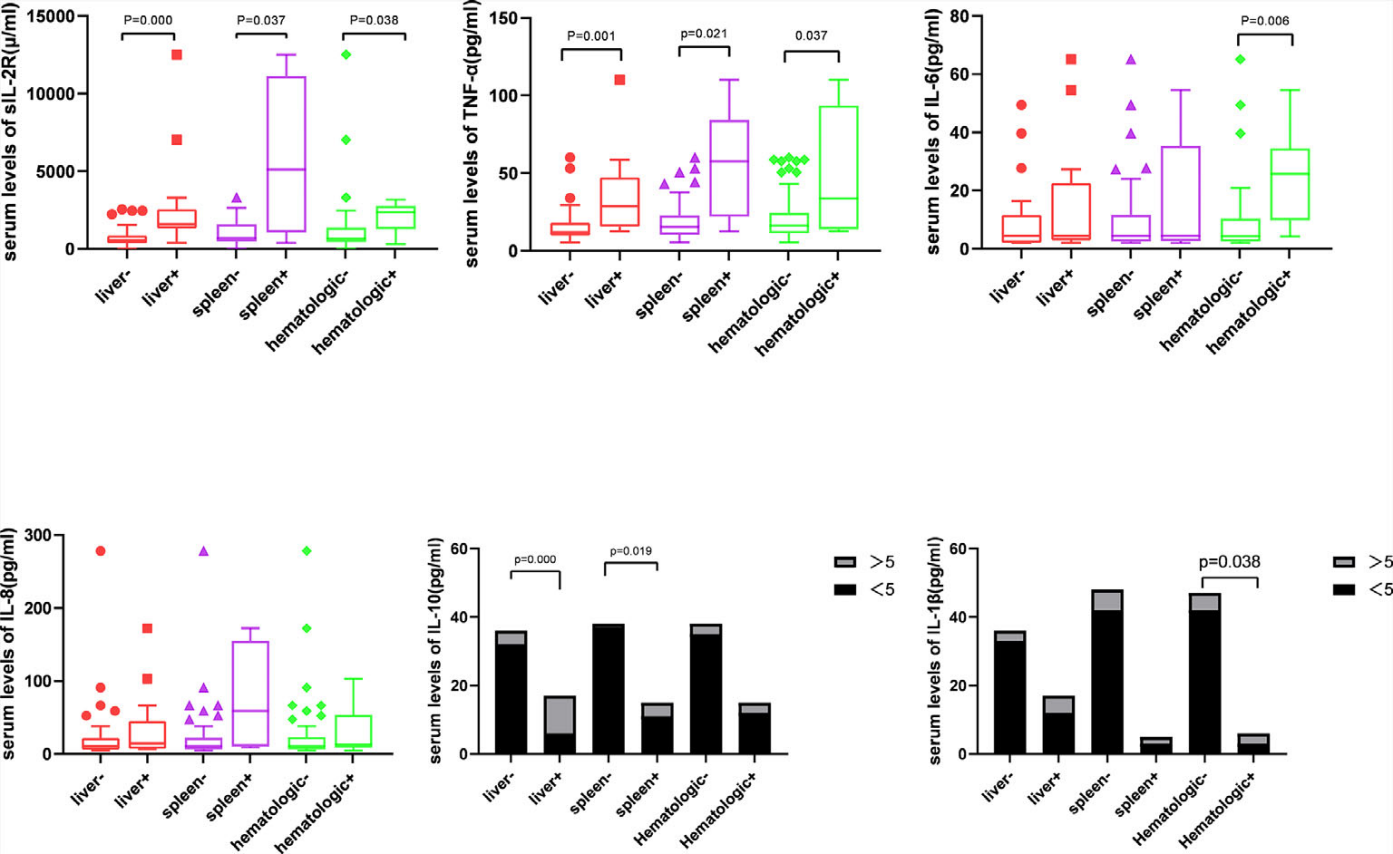
Analysis of inflammatory cytokines and affected organs.

Next, patients were divided into the groups depending on the type of organ involvement. As shown in **Figure 3**, patients with hematologic involvement exhibited a significantly higher sIL-2R, TNF-α, IL-10, and IL-1β expression, as compared to the group without involvement. sIL-2R, TNF-α, and IL-10 were increased in patients with liver or spleen involvement. The percentage of T and Th cells were significantly lower in the spleen+ group, but B cells subset increased by contrast. Similarly, the percentage of Th cells and Th/Ts ratio has decreased in the liver+ group (**Table 3**).

**Table 3:** Lymphocyte subset in different affected organs.

	Affected organs								
	**Liver+**	**Liver-**	**P-value**	**Spleen+**	**Spleen-**	**P-value**	**hematologic+**	**hematologic-**	**P-value**
T cells (%)	57.3 ± 10.6	66.9 ± 6.9	0.001*	56.7 ± 7.7	64.4 ± 9.3	0.058	60.2 ± 14.2	64.3 ± 8.6	0.259
B cells (%)	28.8 ± 12.8	20.0 ± 8.9	0.018*	27.7 ± 10.0	22.6 ± 11.2	0.341	25.4 ± 15.8	22.7 ± 10.4	0.389
Th cells (%)	27.0 ± 7.6	32.5 ± 6.2	0.005*	22.9 ± 3.0	31.5 ± 6.9	0.010*	27.7 ± 8.3	31.2 ± 6.9	0.395
Ts cells (%)	23.0 ± 6.9	26.1 ± 7.7	0.241	27.6 ± 5.0	24.9 ± 7.7	0.333	27.6 ± 12.2	24.8 ± 6.8	0.927
Th/Ts	1.3 ± 0.7	1.4 ± 0.7	0.579	0.9 ± 0.2	1.4 ± 0.7	0.024*	1.2 ± 0.6	1.4 ± 0.7	0.446
CD20+ cells (%)	10.5 ± 10.0	10.6 ± 14.8	0.943	13.8 ± 15.7	10.1 ± 11.3	0.454	19.5 ± 16.9	9.3 ± 10.6	0.165

*Statistical difference between the affected organs groups (P < 0.05).

Th, T helper; Ts, suppressor T.

### Analysis of Immune Indices Before and After Induction Chemotherapy

In order to clarify whether the change in immune indices can be used as a marker of efficacy, serum levels of cytokines, immunoglobulins and lymphocyte subsets were measured prior to treatment. All cases underwent initial chemotherapy of the JLSG-96/02 or CCHG-LCH-2019 regimen, based on disease status. Following the induction of chemotherapy, Ts cells were significantly decreased in non-response group. However, no significant changes were observed in cytokine and immunoglobulin between the response (NAD+AD-B+AD-I) and non-response (AD-S+Worse) groups (**Table 4**). Similarly, no significant changes were observed when we further divided the data into SS, MS, RO- and RO+ sets.

**Table 4:** Comparison of immune indices before and after induction chemotherapy.

Immune index rangeability (After-before)	Response	Non-response	P-value
ΔsIL-2R (µ/ml)	50.9 ± 609.6	13.3 ± 879.0	0.870
ΔIL-6 (pg/ml)	−2.4 ± 18.4	4.3 ± 20.2	0.299
ΔTNF-α (pg/ml)	18.6 ± 66.2	2.9 ± 12.8	0.438
ΔIL-8 (pg/ml)	−0.2 ± 101.0	−31.8 ± 134.4	0.389
ΔIgA (g/l)	0.2 ± 0.7	0.2 ± 0.3	0.963
ΔIgG (g/l)	1.8 ± 4.3	1.3 ± 2.4	0.677
ΔIgM (g/l)	0.3 ± 0.8	0.5 ± 0.3	0.471
ΔIgE (IU/ml)	−13.0 ± 473.2	77.3 ± 158.3	0.539
ΔT cell (%)	−3.4 ± 23.3	−20.4 ± 17.1	0.036*
ΔB cell (%)	2.1 ± 17.5	9.0 ± 11.7	0.150
ΔNK cell (%)	−0.9 ± 8.2	0.9 ± 8.2	0.531
ΔTh cell (%)	−1.7 ± 13.4	−5.9 ± 17.6	0.384
ΔTs cell (%)	−0.2 ± 12.8	−9.6 ± 12.3	0.035*
ΔTh/Ts	0.5 ± 3.3	−0.03 ± 1.0	0.347
ΔCD20+ cell (%)	2.7 ± 16.9	13.2 ± 10.5	0.153

Th, T helper; Ts, suppressor T. *Statistical difference between the Affected organs groups (P < 0.05).

## Discussion

LCH is a heterogeneous disease and it can affect from single, localized lesions to multiple systems/organs, including risk organs. The prognosis of different subtypes of LCH is highly variable. Partial cases with a solitary bone lesion can be cured by curettage, and children with skin-isolated LCH usually require no specific therapy, as spontaneous healing may occur. MS-LCH with risk organ involvement, such as liver and hematopoietic system, has a poor response to therapy, resulting in a particularly dismal prognosis (14). To date, the pathogenesis of LCH remains unclear. In recent years, it was found that the MAPK pathway is involved in the pathogenesis of LCH, with the mutation of more than half of BRAF, 20% of MAP2K1 and rare ARAF, MAP3K1 (2, 15). However, studies have shown that cytokines are essential for local infiltration and metastasis of LCH cells (16, 17). De Graaf et al. found a variety of cytokines in LCH lesions, such as IL-1, TGF-α, TGF-β, GM-CSF, TNF-α, and TFN-γ (11). Kannourakis et al. extracted and cultured monocytes from eosinophilic granulomatous tissues in patients with LCH, and found that such monocytes could produce a large number of IL-1, TNF-α, GM-CSF, IL-8, and LIF-21 (12). Pathologically, one of the cardinal manifestations of LCH is the accumulation of pathologic LCH cells in target tissues, surrounding by the varying degrees of lymphocyte infiltration, such as T cells, macrophages, eosinophils, and B cells, as well as multinucleated giant cells. Egeler used immunohistochemical techniques to detect LCH and T cells, macrophages and eosinophils in 14 children with LCH; it was found that cytokines mainly originated from LCH and T cells (18). On the other hand, LCH is seen as a result of a misguided differentiation of myeloid dendritic cell (DC) precursors originating from multiple hematopoietic stem cells, whose differentiation, maturation and migration are regulated by diverse cytokines (19). For example, Cumberbatch found that DCs were significantly concentrated in the lymph nodes of mice following a subcutaneous injection of TNF-α. However, this phenomenon did not occur after injecting the same dose of TNF-α directly into the lymph nodes, suggesting that TNF-α may contribute to the migration of DC/LC lineage cells (20).

In 1994, Kannourakis reported elevated peripheral blood levels of GM-CSF and IL-3 in MS-LCH patients, recognizing that cytokines in LCH lesions were likely to be released to the circulation (12). Our results demonstrated that the serum levels of sIL-2R, TNF-α, and IL-10 in the MS-LCH patients with RO+ were significantly higher than SS-LCH patients. In particular, sIL-2R, TNF-α, and IL-10 were noticeably increased in patients with liver spleen and hematologic involvement. This indicated that serum levels of sIL-2R, TNF-α, and IL-10 may reflect the severity of the disease in LCH to a certain extent. Morimoto’s study, which found the serum levels of 9 humoral factors, including IL-2R, IL-8, IL-18, and M-CSF, substantially higher in patients with MS-LCH than in those with SS-LCH (21). sIL-2R consists of three chains; α (also termed IL-2Rα, CD25, or Tac antigen), β (also termed IL-2Rβ or CD122), and γ (also termed IL-2Rγ or CD132). The principal functions of IL-2Rα are to bind with IL-2 and promote optimal IL-2 signaling through its association with the IL-2Rβ and IL-2Rγ chains, while inhibiting the clonal proliferation of activated T cells. IL-2, IL-2Rβ, and IL-2Rγ are rapidly degraded, but IL-2Rα is recycled to the cell surface. Thus, the available concentration of the soluble form of IL-2R (sIL2Rα) determines the tempo, magnitude and extent of T cell immune responses. TNF-α is mainly produced by activated macrophages with a wide range of biological functions, including the induction of inflammation, anti-tumor effect, activation of T cells, and mediated immune response. IL-2R and TNF-α have been reported to play an important role in inducing the generation and maturation of LCs *in vitro (* 22). It has also been found that serum sIL-2R and TNF-α are significantly elevated in LCH patients (23, 24). IL‐10 could bind with IL‐10 receptors on tumor cells to activate STAT3, which thus promotes the proliferation of tumor cells *via* the activation of cell cycle‐related proteins (25, 26). It has been hypothesized that IL-10 may play a role in the assessment of LCH, since a study reported an increased expression of IL-10 in LCH lesions (27).

Cytokine elevation indicates a disturbance in cellular immunity. The number of T cells in an active state in LCH lesions was second only to the number of LCH cells (27). Treg cells were also found to be increased in the peripheral blood of LCH patients (28, 29). Our findings have shown that the percentage of Th cells and Th/Ts ratio in the peripheral blood of LCH patients with liver or spleen involvement was lower than those without involvement. After that, the level of Ts cell dropped during the induction treatment in non-response group. Suppressor T cell (Ts), also call regulatory cells (Tregs), were the second most common type of infiltrating immune cell in LCH tissue (30). Ts could inhibit immune responses against LCH cells, which lead to increasing survival of LCH cells, granuloma maintenance, and dissemination (28). It seems to reflect that the abnormal regulation of T lymphocyte may affect the disease progression.

The present study failed to reflect the effects of the changes in immune indicators on the assessment of efficacy. Some clinical observation showed that the ratio of serum TNF-α (23), IL-2R, RANKL, OPG, and SRANKL/OPG (24) significantly decreased following chemotherapy. Patients with IL-18 serum levels of >500 pg/ml were insensitive to JLSG treatment (21). To date, there have been a number of studies with similar results, at home and abroad, but it is necessary to explore and confirm the underlying mechanisms by a large-scale, multicenter trial. Therefore, changes in serum cytokines may be used as a marker of the curative effect of clinical treatment, but whether it is a sensitive and specific marker requires further research.

Children with LCH often present with MS damage at onset and frequently involved risk organs. These cases are characterized by a long disease course, low cure rate and easy recurrence. Increased understanding of the pathogenesis and pathological changes of different clinical types of LCH will help optimize and personalize therapy, which can, in turn, improve the curative effect. Initial reports have indicated that immunocyte and cytokine immunoregulatory disorders might be linked to the occurrence, and development of LCH. Pertinent laboratory inspections can be used as prognostic indices for children with LCH, to improve risk-stratification and guide immunotherapy. Besides, the short assessment time, the change of the clinical classification and complications, such as infections during treatment may also affect the results. It may be possible to collect immune indicators of patients during the 12 weeks of induction and maintenance treatment in a follow-up study.

## Data Availability Statement

The original contributions presented in the study are included in the article/supplementary mterial. Further inquiries can be directed to the corresponding authors.

## Ethics Statement

The studies involving human participants were reviewed and approved by Sun Yat-Sen Memorial Hospital. Written informed consent to participate in this study was provided by the participants’ legal guardian/next of kin.

## Author Contributions

YL: conceptualization, writing—review and editing and supervision. HK: conceptualization, methodology, and supervision. CF: formal analysis, writing—original draft, and visualization. XP: data curation. HG: validation. LZ: data curation. XX: formal analysis. WW: supervision. JL: data curation. JF: supervision. All authors contributed to the article and approved the submitted version.

## Conflict of Interest

The authors declare that the research was conducted in the absence of any commercial or financial relationships that could be construed as a potential conflict of interest.

The handling editor declared a shared affiliation, though no other collaboration, with several of the authors CF, YL, HK, XP, HG, LZ, XX, WW, JL, and JF.
